# The role of health-related behavioural factors in accounting for inequalities in coronary heart disease risk by education and area deprivation: prospective study of 1.2 million UK women

**DOI:** 10.1186/s12916-016-0687-2

**Published:** 2016-10-13

**Authors:** Sarah Floud, Angela Balkwill, Kath Moser, Gillian K. Reeves, Jane Green, Valerie Beral, Benjamin J. Cairns, for the Million Women Study Collaborators, Emily Banks, Valerie Beral, Lucy Carpenter, Carol Dezateux, Jane Green, Julietta Patnick, Richard Peto, Cathie Sudlow, Hayley Abbiss, Simon Abbott, Miranda Armstrong, Krys Baker, Angela Balkwill, Isobel Barnes, Valerie Beral, Judith Black, Kathryn Bradbury, Anna Brown, Benjamin Cairns, Dexter Canoy, Andrew Chadwick, Dave Ewart, Sarah Ewart, Lee Fletcher, Sarah Floud, Toral Gathani, Laura Gerrard, Adrian Goodill, Jane Green, Lynden Guiver, Alicia Heath, Michal Hozak, Carol Hermon, Isobel Lingard, Sau Wan Kan, Nicky Langston, Kath Moser, Kirstin Pirie, Gillian Reeves, Keith Shaw, Emma Sherman, Helena Strange, Sian Sweetland, Sarah Tipper, Claire Wotton, Lucy Wright, Owen Yang, Heather Young

**Affiliations:** Cancer Epidemiology Unit, Nuffield Department of Population Health, University of Oxford, Richard Doll Building, Old Road Campus, Roosevelt Drive, Oxford, OX3 7LF UK

**Keywords:** Area deprivation, Coronary heart disease, Education, Health behaviour, Socio-economic status, Women

## Abstract

**Background:**

Some recent research has suggested that health-related behaviours, such as smoking, might explain much of the socio-economic inequalities in coronary heart disease (CHD) risk. In a large prospective study of UK women, we investigated the associations between education and area deprivation and CHD risk and assessed the contributions of smoking, alcohol consumption, physical activity and body mass index (BMI) to these inequalities.

**Methods:**

After excluding women with heart disease, stroke or cancer at recruitment, 1,202,983 women aged 56 years (SD 5 years) on average, were followed for first coronary event (hospital admission or death) and for CHD mortality. Relative risks of CHD were estimated by Cox regression, and the extent to which any association could be accounted for by smoking, alcohol, physical inactivity, and BMI was assessed by calculating the percentage reduction in the relevant likelihood-ratio (LR) statistic after adjustment for these factors, separately and together.

**Results:**

A total of 71,897 women had a first CHD event (hospital admission or death) and 6032 died from CHD during 12 years follow-up. In analyses adjusted by age, birth cohort and region of residence only, lower levels of education and greater deprivation were associated with higher risks of CHD (*P*_heterogeneity_ < 0.0001 for each); associations for education were found within every level of deprivation and for deprivation were found within every level of education. Smoking, alcohol consumption, physical inactivity and BMI accounted for most of the associations (adjustment for all four factors together reduced the LR statistics for education and for deprivation by 76 % and 71 %, respectively, for first CHD event; and by 87 % and 79 %, respectively, for CHD mortality). Of these four factors, adjustment for smoking resulted in the largest reduction in the LR statistic. Given the large reduction in the predictive values of education and deprivation after adjustment for only four health-related behavioural factors recorded just at recruitment, residual confounding might plausibly account for the remaining associations.

**Conclusions:**

Most of the association between CHD risk and education and area deprivation in UK women is accounted for by health-related behaviours, particularly by smoking and to a lesser extent by alcohol consumption, physical inactivity and BMI.

**Electronic supplementary material:**

The online version of this article (doi:10.1186/s12916-016-0687-2) contains supplementary material, which is available to authorized users.

## Background

Inequalities in coronary heart disease (CHD) risk have been reported for women in relation to both education and area deprivation, with women who are less educated or more deprived being more likely to develop, and die from, heart disease [1–21]. Most of the evidence regarding education comes from the US and Scandinavia, which historically have had different education systems from the UK in terms of length of compulsory schooling [22–24], and there have been relatively few studies conducted on women in the UK [14, 16, 19]. Previous findings suggest that much of the association between low education and higher CHD risk, as well as that between greater deprivation of an area and higher CHD risk, is mediated through factors such as current smoking, alcohol consumption, inactive lifestyles, and obesity (all of which we refer to here as “health-related behaviours”, since obesity is largely a marker of behaviours such as dietary intake and physical inactivity) [2, 4, 7, 12, 25–28]. The extent to which these health-related behaviours explain socioeconomic inequalities in CHD risk, and the relative importance of the different behaviours, is unclear given that previous estimates have varied between studies and some behaviours, such as alcohol consumption and physical inactivity, have rarely been examined as explanatory factors [16].

The Million Women Study is a large contemporary cohort of women in the UK, approximately a fifth of whom have smoked for their entire adult lives and over half of whom are overweight or obese [29, 30]. Our aim was to examine the associations between education and area deprivation and risk of CHD in this cohort, and to investigate the extent to which smoking, alcohol consumption, physical inactivity and body mass index (BMI) can, separately and jointly, account for the associations.

## Methods

### Data collection

The Million Women Study is a population-based prospective study of women in the UK. Details of the design and methods of the study have been described elsewhere [31]. Briefly, 1.3 million women were invited for breast cancer screening at National Health Service (NHS) clinics in England and Scotland and were recruited to the study between 1996 and 2001 by completing a questionnaire. The respondents gave written consent to participate and ethical approval was provided by the Oxford and Anglia Multi-Centre Research Ethics Committee. Study questionnaires and further details of the data and access policies can be viewed on the study website [32].

### Follow-up

Individuals in the study were linked electronically by their unique NHS number and date of birth to routinely collected NHS data through which they were followed-up for deaths, emigrations, cancer registrations and hospital admissions. Information is provided on the date of each event, with diagnoses coded to the World Health Organization’s International Classification of Diseases 10th Revision (ICD-10).

The main outcomes for these analyses were first CHD event and CHD mortality (ICD-10: I20-I25). A first CHD event was defined as a first hospital admission with CHD diagnosis (in any position) or death with CHD as the underlying cause, whichever came first. CHD mortality was defined as death with CHD as the underlying cause. In a sample of this cohort, we have shown that 92 % of hospital admission diagnoses coded to ICD-10: I20-I25 were confirmed as CHD by primary care physicians [33].

Women were followed from recruitment until 31 March 2011 in England and 31 December 2008 in Scotland because hospital admission data were incomplete after these dates. Person-years were calculated from recruitment until the date of hospital admission for CHD, death, emigration or end of follow-up, whichever came first. Follow-up is virtually complete, with only 1 % having been lost to follow-up and contributing person-years up to the date of loss. Women were excluded from the analyses if they had previous heart disease, stroke or cancer (except non-melanoma skin cancer) and if there was no information on either education or area deprivation. The remaining 1,202,983 women formed the population at risk for these analyses.

### Education and area deprivation

Level of education was determined using the reported age at leaving school and the highest educational qualification achieved. Education was divided into five categories: tertiary qualifications (college or university), secondary qualifications (A levels or O levels usually obtained at 18 and 16 years of age, respectively), technical qualifications (nursing, teaching, clerical or commercial), completed compulsory schooling with no qualifications, and did not complete compulsory schooling (with no qualifications). There was a change in the compulsory school leaving age from 14 to 15 years old on April 1, 1947, in England and Scotland; whether participants left school before the compulsory leaving age which applied to them was calculated from this date, the age at which participants reported leaving school, and their date of birth.

Area deprivation level was determined for each participant from reported postcode at recruitment within the smallest geographical unit to which a Townsend score [34] could be assigned (census enumeration districts in England, census output areas in Scotland) and was categorised by tertiles and quintiles before any exclusions were made for this analysis. The Townsend index is constructed from four census variables: percentage of households without a car, percentage of overcrowded households, percentage of households not owner-occupied and percentage of persons unemployed. Compared to national data, the women in the Million Women Study are less socio-economically deprived than the UK average, but all levels of deprivation are represented [35].

In a follow-up questionnaire, which 521,170 participants completed, on average, 12.5 years (SD 0.5 years) after recruitment, deprivation in childhood was assessed using questions on household characteristics when the women were about 10 years old, including housing tenure (rented, owned/mortgaged, other), availability of household plumbing (running hot water, indoor toilet) and number of people in their bedroom. On average, these women were 10 years old in 1952.

### Statistical analysis

All analyses used Stata 13.1 (StataCorp, College Station, TX, USA). Cox regression models were used to estimate hazard ratios (called relative risks (RRs) here) and 95 % confidence intervals for risk of first CHD events and of CHD mortality by education and area deprivation. The underlying time variable was attained age and models were stratified by region of residence at recruitment (10 geographic regions). The models were also stratified by birth cohort (born before 1939, born 1939–1945, born after 1945) with similar numbers of women in each birth cohort category, and which reflect the potential influence of societal changes related to World War II on CHD risk. We examined the effect of adjusting for four self-reported health-related behaviours: cigarette smoking (never, past, current < 15 per day, current ≥ 15 per day), alcohol consumption (0, < 7, 7–14, ≥ 15 drinks per week), physical activity (strenuous exercise “enough to cause sweating or a fast heart beat” rarely/never, less than once per week, more than once per week) and BMI (< 22.5, 22.5–24.9, 25.0–27.4, 27.5–29.9, ≥ 30 kg/m^2^). Adjustment was made for each of these health-related behaviour variables separately and then for all four simultaneously.

Models for education and area deprivation were adjusted for each other, since they were only moderately correlated (Spearman’s rho = 0.23, *P* < 0.0001). We also cross-classified women into 3 × 3 categories based on level of education (tertiary, secondary/technical, no qualifications) and area deprivation (least deprived third, middle third, most deprived third).

In this report, the likelihood-ratio (LR) χ^2^ statistics quoted in the text and tables provide a quantitative measure of the extent to which education and area deprivation predict CHD risk in different models (e.g. with and without adjustment for particular health-related behaviours). If associations of CHD risk with education or area deprivation are explained wholly or in part by particular health-related behaviours, then the associated LR statistics will be smaller in models which include the health-related behaviours than in models which do not. Changes in the LR statistics between models which do and do not adjust for health-related behaviours are therefore a measure of the extent to which the behaviours account for any associations between CHD risk and education or area deprivation [36]. Percent reductions in the LR statistics for education and area deprivation were calculated for a series of models which included each of the four health-related behaviours, singly and then jointly.

In this cohort, we have shown good validity of smoking (against measured cotinine levels) [37] and BMI (against measured height and weight) [38], as well as good repeatability of reported alcohol intake and physical activity between recruitment and the re-survey three years later [39, 40]. Among 19,309 women who completed the recruitment questionnaires twice, the repeatability of reporting of each of the four health-related behaviours considered here did not vary by education or by deprivation (Additional file 1: Table S1). We investigated possible differential changes by education and deprivation level in the four health-related behaviours over time, by examining changes in these behaviours from recruitment to the re-survey questionnaire 3 years later.

In four separate sensitivity analyses, we (1) restricted analyses to women who reported never smoking in order to assess the extent of residual confounding by smoking; (2) categorised deprivation based on quintiles of the national distribution of deprivation for comparison with national statistics; (3) excluded women who reported at recruitment that they were being treated for hypertension and diabetes, as these factors may be mediators of any observed associations; and (4) we allowed for intra-group correlations within census enumeration districts using a clustered sandwich estimator [41].

## Results

Women with no educational qualifications were more likely to live in deprived areas and tended to be older and of shorter stature, on average, than those with qualifications; they were also more likely to smoke, be obese, be physically inactive, and drink less alcohol (Table 1). Similarly, women living in the most deprived areas had fewer educational qualifications and were of shorter stature than those living in the most affluent areas, and were also more likely to smoke, be obese, physically inactive, and drink less alcohol.

**Table 1:** Characteristics of 1,202,983 women by education and by area deprivation

	Education			Area deprivation			All women
Characteristics	Tertiary	Secondary/Technical	No qualifications	Least deprived third	Middle third	Most deprived third	
N (%)	161,030 (13)	522,465 (43)	519,488 (43)	412,757 (34)	403,223 (34)	387,003 (32)	1,202,983
Most deprived third, %	21	24	43	–	–	–	32
No qualifications, %	–	–	–	31	41	58	43
Mean age, years (SD)	55.4 (4.7)	55.7 (4.7)	56.5 (4.8)	55.9 (4.8)	56.0 (4.8)	56.0 (4.8)	56.0 (4.8)
Mean height, cm (SD)	163.5 (6.6)	162.5 (6.5)	161.1 (6.8)	162.5 (6.5)	162.3 (6.7)	161.3 (6.9)	162.0 (6.7)
Current smoker %	11	16	27	14	18	29	20
Mean alcohol, drinks/week (SD)	5.7 (6.0)	4.6 (5.5)	3.2 (4.7)	4.6 (5.4)	4.2 (5.3)	3.6 (5.1)	4.2 (5.3)
Physically inactive %	31	41	60	41	46	57	48
Mean body mass index, kg/m^2^ (SD)	25.3 (4.3)	25.8 (4.4)	26.7 (4.8)	25.6 (4.2)	26.0 (4.5)	26.8 (5.0)	26.1 (4.6)
Diabetes %	1	2	3	2	2	3	2
Hypertension %	11	14	17	13	14	16	15
Follow-up							
First CHD events	5900	25,630	40,367	19,204	22,455	30,238	71,897
Person years (1000s)	1867	6067	5978	4820	4696	4397	13,912
CHD deaths	415	1910	3707	1342	1810	2880	6032
Person years (1000s)	1893	6180	6158	4905	4795	4530	14,230

Some percentages do not add up to 100 due to rounding

Indicators of childhood deprivation were, as expected, associated with lower levels of education and greater area deprivation in adulthood (Table 2). Women who, as adults, had no educational qualifications and who lived in deprived areas were more likely to have lived in rented housing, to have had no indoor plumbing, and to have shared a bedroom when they were 10 years old (which was in 1952, on average). Deprivation in childhood was more strongly associated with lack of educational qualifications than with deprivation in adulthood, as evidenced by the much larger χ^2^ values for heterogeneity for the associations with education than the associations with deprivation (Table 2).

**Table 2:** Household circumstances at age 10 by subsequent education and area deprivation

		Household circumstances at age 10		
	N (%)	House was rented, %	No indoor plumbing^a^, %	Shared bedroom, %
Education				
Tertiary	96,681 (19)	40	10	41
Secondary/technical	255,961 (49)	54	16	47
No qualifications	168,528 (32)	71	26	60
χ^2^ for heterogeneity		24,348	10,960	10,434
Area deprivation				
Least deprived third	200,694 (39)	54	16	48
Middle third	180,550 (35)	56	17	49
Most deprived third	139,926 (27)	63	19	54
χ^2^ for heterogeneity		2755	862	1406

In 521,170 women who completed the third follow-up questionnaire about 12 years after recruitment

Some percentages do not add up to 100 due to rounding

Percentages were adjusted by age at time of reporting household circumstances

^a^No indoor plumbing was defined as neither running hot water nor indoor toilet

During an average follow-up period of 12 years per woman (11.6 years (SD 2.3 years) for CHD incidence, and 11.8 years (SD 1.9 years) for CHD mortality), there were 71,897 first CHD events and, overall, 6032 women died of CHD (Table 1).

### Education and CHD

With minimal adjustment (for age, birth cohort and region only), there were clear differences in CHD incidence and mortality between women with different levels of education (Table 3). For example, women who had completed compulsory schooling with no qualifications had almost twice the risk of a first coronary event as women with tertiary qualifications. Similar differences were observed for risk of death from CHD (Table 3). Simultaneous adjustment for the four health-related behaviours, smoking, alcohol consumption, physical inactivity and BMI, strongly attenuated the relative risk estimates and the LR statistics for the associations declined by 76 % for first CHD event and by 87 % for CHD death. Adjustment for smoking alone had the greatest effect, with the LR statistic declining by 35 % for first CHD event, and by 55 % for CHD death. When the minimally-adjusted associations with education were adjusted for area deprivation alone, the LR statistic declined by 38 % for first CHD event. However, after adjustment for all four health-related behaviours, the additional adjustment for area deprivation had only a small effect on the risk estimates and the LR statistic declined by just 7 %, suggesting that the contribution of deprivation to the association between education and CHD risk is largely accounted for by the four health-related behaviours.

**Table 3:** Relative risks and 95 % confidence intervals (CI) of coronary heart disease (CHD) incidence and CHD mortality by education

Education	Tertiary	Secondary	Technical	No qualifications		LR^a^	% reduction in LR^a^
				Compulsory schooling	< Compulsory schooling		
No. of women	161,030	318,635	203,830	491,521	27,967		
CHD INCIDENCE							
No. of first CHD events	5900	14,414	11,216	37,516	2851		
Relative risk (95 % CI), adjusted for:							
Age, birth cohort, region only	1.00 (–)	1.23 (1.19–1.27)	1.42 (1.38–1.47)	1.92 (1.86–1.97)	2.46 (2.35–2.57)	4262	–
Age, birth cohort, region, deprivation	1.00 (–)	1.21 (1.18–1.25)	1.39 (1.34–1.43)	1.73 (1.68–1.77)	2.13 (2.04–2.23)	2653	38
Age, birth cohort, region, smoking	1.00 (–)	1.19 (1.15–1.23)	1.35 (1.31–1.40)	1.70 (1.66–1.75)	2.15 (2.06–2.25)	2757	35
Age, birth cohort, region, alcohol	1.00 (–)	1.21 (1.17–1.24)	1.37 (1.33–1.41)	1.76 (1.71–1.81)	2.18 (2.09–2.28)	2963	31
Age, birth cohort, region, physical inactivity	1.00 (–)	1.20 (1.16–1.24)	1.36 (1.32–1.40)	1.76 (1.71–1.81)	2.23 (2.13–2.34)	3086	28
Age, birth cohort, region, body mass index	1.00 (–)	1.21 (1.17–1.25)	1.38 (1.34–1.42)	1.79 (1.74–1.84)	2.26 (2.16–2.37)	3353	21
Age, birth cohort, region, health behaviours^b^	1.00 (–)	1.13 (1.10–1.17)	1.23 (1.19–1.27)	1.41 (1.37–1.45)	1.71 (1.64–1.79)	1041	76
Age, birth cohort, region, health behaviours,^b^plus deprivation	1.00 (–)	1.13 (1.09–1.16)	1.22 (1.18–1.26)	1.34 (1.31–1.38)	1.61 (1.54–1.68)	722	83
CHD MORTALITY							
No. of CHD deaths	415	1104	806	3415	292		
Relative risk (95 % CI), adjusted for:							
Age, birth cohort, region only	1.00 (–)	1.34 (1.20–1.50)	1.41 (1.25–1.58)	2.34 (2.12–2.60)	3.23 (2.78–3.75)	626	–
Age, birth cohort, region, deprivation	1.00 (–)	1.31 (1.17–1.47)	1.35 (1.20–1.52)	1.98 (1.79–2.20)	2.58 (2.21–3.00)	351	44
Age, birth cohort, region, smoking	1.00 (–)	1.23 (1.10–1.38)	1.25 (1.11–1.40)	1.78 (1.60–1.97)	2.34 (2.01–2.72)	281	55
Age, birth cohort, region, alcohol	1.00 (–)	1.31 (1.17–1.47)	1.35 (1.20–1.52)	2.06 (1.86–2.29)	2.68 (2.31–3.12)	408	35
Age, birth cohort, region, physical inactivity	1.00 (–)	1.28 (1.14–1.43)	1.29 (1.15–1.45)	1.99 (1.80–2.21)	2.68 (2.30–3.11)	399	36
Age, birth cohort, region, body mass index	1.00 (–)	1.32 (1.18–1.48)	1.37 (1.22–1.55)	2.20 (1.99–2.44)	2.96 (2.54–3.44)	517	17
Age, birth cohort, region, health behaviours^b^	1.00 (–)	1.16 (1.04–1.30)	1.10 (0.98–1.24)	1.36 (1.23–1.51)	1.70 (1.45–1.98)	80	87
Age, birth cohort, region, health behaviours,^b^plus deprivation	1.00 (–)	1.15 (1.03–1.29)	1.09 (0.97–1.23)	1.28 (1.15–1.42)	1.55 (1.33–1.81)	48	92

^a^Likelihood-ratio test statistic

^b^Smoking, alcohol consumption, physical inactivity, body mass index

### Area deprivation and CHD

Gradients in CHD incidence and mortality were also found by level of area deprivation (Table 4). With minimal adjustment (for age, birth cohort and region only), women in the most deprived quintile had twice the risk of a first CHD event and three times the risk of CHD death than women in the least deprived quintile. As with education, simultaneous adjustment for all four health-related behaviour variables led to substantial attenuations of the risk estimates and the LR statistics declined by 71 % for first CHD event and 79 % for CHD mortality with adjustment for all four variables. Again adjustment for smoking had the greatest effect (a reduction in the LR statistic of 41 % for first CHD event and 56 % for CHD death).

**Table 4:** Relative risks and 95 % confidence intervals (CIs) of coronary heart disease (CHD) incidence and CHD mortality by area deprivation

Area deprivation	Least deprived quintile	Q2	Q3	Q4	Most deprived quintile	LR^a^	% reduction in LR^a^
No. of women	248,286	244,341	242,627	239,401	228,328		
CHD INCIDENCE							
No. of first CHD events	11,052	12,309	13,365	15,429	19,742		
Relative risk (95 % CI), adjusted for:							
Age, birth cohort, region only	1.00 (–)	1.12 (1.09–1.15)	1.24 (1.21–1.27)	1.46 (1.43–1.50)	1.96 (1.92–2.01)	4147	–
Age, birth cohort, region, education	1.00 (–)	1.09 (1.06–1.12)	1.18 (1.15–1.21)	1.35 (1.32–1.39)	1.72 (1.67–1.76)	2538	39
Age, birth cohort, region, smoking	1.00 (–)	1.09 (1.07–1.12)	1.19 (1.16–1.22)	1.36 (1.32–1.39)	1.70 (1.66–1.74)	2461	41
Age, birth cohort, region, alcohol	1.00 (–)	1.11 (1.08–1.13)	1.21 (1.18–1.24)	1.40 (1.37–1.44)	1.82 (1.77–1.86)	3171	24
Age, birth cohort, region, physical inactivity	1.00 (–)	1.11 (1.08–1.14)	1.21 (1.18–1.25)	1.41 (1.38–1.45)	1.84 (1.80–1.89)	3348	19
Age, birth cohort, region, body mass index	1.00 (–)	1.10 (1.08–1.13)	1.21 (1.18–1.24)	1.40 (1.37–1.44)	1.84 (1.79–1.88)	3352	19
Age, birth cohort, region, health behaviours^b^	1.00 (–)	1.06 (1.04–1.09)	1.12 (1.10–1.15)	1.23 (1.20–1.26)	1.45 (1.42–1.49)	1187	71
Age, birth cohort, region, health behaviours,^b^plus education	1.00 (–)	1.05 (1.03–1.08)	1.10 (1.07–1.13)	1.20 (1.17–1.23)	1.38 (1.35–1.41)	867	79
CHD MORTALITY							
No. of CHD deaths	727	965	1045	1357	1938		
Relative risk (95 % CI), adjusted for:							
Age, birth cohort, region only	1.00 (–)	1.32 (1.20–1.46)	1.45 (1.32–1.60)	1.92 (1.75–2.10)	2.91 (2.67–3.17)	814	–
Age, birth cohort, region, education	1.00 (–)	1.28 (1.16–1.41)	1.36 (1.24–1.50)	1.73 (1.58–1.90)	2.45 (2.24–2.67)	539	34
Age, birth cohort, region, smoking	1.00 (–)	1.25 (1.14–1.38)	1.31 (1.20–1.45)	1.60 (1.46–1.76)	2.09 (1.91–2.28)	362	56
Age, birth cohort, region, alcohol	1.00 (–)	1.30 (1.18–1.43)	1.39 (1.27–1.53)	1.79 (1.63–1.96)	2.56 (2.35–2.79)	610	25
Age, birth cohort, region, physical inactivity	1.00 (–)	1.30 (1.18–1.43)	1.40 (1.28–1.54)	1.80 (1.65–1.97)	2.60 (2.38–2.83)	636	22
Age, birth cohort, region, body mass index	1.00 (–)	1.31 (1.19–1.44)	1.41 (1.28–1.55)	1.83 (1.67–2.01)	2.69 (2.47–2.94)	691	15
Age, birth cohort, region, health behaviours^b^	1.00 (–)	1.21 (1.10–1.33)	1.22 (1.11–1.34)	1.40 (1.28–1.54)	1.68 (1.54–1.84)	168	79
Age, birth cohort, region, health behaviours,^b^plus education	1.00 (–)	1.20 (1.09–1.32)	1.20 (1.09–1.32)	1.37 (1.25–1.50)	1.61 (1.47–1.76)	135	83

^a^Likelihood-ratio test statistic

^b^Smoking, alcohol consumption, physical inactivity, body mass index

From recruitment to the re-survey 3 years later, women with some educational qualifications were slightly more likely to quit smoking but slightly less likely to put on weight and to reduce their alcohol intake than women with no qualifications (Additional file 1: Table S2). Similar small differences were also seen by area deprivation (Additional file 1: Table S2). These differences would contribute to residual confounding by these factors.

When education and area deprivation were cross-classified, the risk estimates for the association with first CHD event were attenuated after adjustment for the four lifestyle factors and the LR statistic reduced by 74 % as had been found in the main analyses when education and area deprivation were not combined (Additional file 1: Table S3). In a sensitivity analysis restricted to never smokers, the relative risks for CHD in relation to education were lower than those found after adjusting for smoking in the whole cohort, suggesting that there may still be residual confounding by smoking (Additional file 1: Tables S4 and S5). The sensitivity analyses in which area deprivation was categorised based on quintiles of the national distribution (Additional file 1: Table S6), the one excluding women treated for hypertension and diabetes (Additional file 1: Tables S7 and S8), and the one allowing for intra-group correlations (Additional file 1: Table S9), all showed similar results to the main findings.

## Discussion

In this large prospective study of UK women, education and area deprivation were each strongly associated with CHD risk in analyses that were minimally adjusted only for age, year of birth and region of residence. However, four health-related behaviours, smoking, alcohol consumption, physical inactivity and BMI accounted for most of the inequalities observed for CHD risk both by education and by area deprivation. Smoking had the greatest single effect in attenuating the risks. Overall, at least 70 % of the variation in risk by education and area deprivation was accounted for by adjustment for these four health-related behaviours recorded at recruitment. Given that only four health-related behaviours were included in the model and that they are an imperfect measure of behaviours throughout the follow-up period, residual confounding could plausibly account for any remaining associations [42]. Indeed, re-measurement 3 years after recruitment showed that changes in these health-related behaviours varied somewhat by education and by deprivation, providing supporting evidence that some residual confounding is likely.

Prospective evidence on educational inequalities and CHD risk for women is limited. Our findings are consistent with findings from another UK study, where the same four factors as well as other socioeconomic factors and prior disease appeared to account for about 70 % of the association between age at leaving full time education and CHD risk [14]. Similar findings were reported from a prospective study in Sweden [4]. Although somewhat smaller reductions were found in Norway and Finland, the Norwegian study did not adjust for BMI and the Finnish study did not adjust for physical activity or alcohol consumption [7, 8].

Area deprivation is an indicator of the general characteristics of the people who live in the area in which participants live. We found that the influence of area deprivation on CHD risk also appears to be substantially mediated through health-related behaviours, consistent with previous evidence [19, 26]. For example, in a record linkage study of UK women, associations between area deprivation and CHD risk were reported to be substantially attenuated after adjustment for age, smoking, BMI, diabetes, blood pressure, cholesterol levels and medication use [19].

In the present study, smoking was the single strongest confounding factor, as it accounted for the largest proportion of the associations of education and deprivation with CHD risk. Smoking has previously been estimated to account for around half of socioeconomic inequalities in overall mortality for men [43]. UK smoking rates are higher in the socioeconomically disadvantaged [44] and smoking is known to be an important cause of CHD [29]. Alcohol consumption, physical inactivity and BMI each accounted for a proportion of the association with CHD risk, but generally not as much as that attributed to smoking. In analyses restricted to non-smokers, the adjusted risk estimates were lower than in the main analyses, providing further evidence that there may well be residual confounding by smoking.

Education may influence health-related behaviours by promoting a greater awareness of what constitutes healthy lifestyle behaviours [45, 46] and, at the same time, by leading to higher earnings which might affect the ability to lead a healthy lifestyle [47–49]. The social norms of an area have been found to influence the acceptability of smoking [27], and features of the physical environment, such as the availability of places to exercise and healthy food stores, may also affect the ability to lead healthy lives [26, 28]. It has been proposed that psycho-social factors related to poverty could have a direct effect on health outcomes [50], but the fact that the associations between CHD risk and education and deprivation appeared to be largely due to smoking, alcohol consumption, physical inactivity and BMI suggests that any direct psycho-social effects on CHD risk might be relatively minor. A similar conclusion can be drawn from an analysis of the UK Whitehall II study, which found that the association between employment grade and cardiovascular mortality was substantially accounted for by adjustment for health behaviours [51]. Nevertheless, psycho-social factors could affect behaviours [51]; for example, smoking can be used as a coping strategy for stress [52]. We were unable to investigate other measures of socioeconomic status, such as those based on occupation, although women’s own occupations can mask the extent of social inequality unless augmented by information about their husbands’ occupations and income [53].

We did not investigate possible inequality in uptake of treatments for CHD, which might have affected the associations of education and area deprivation with CHD mortality, but no social gradient in uptake of treatment in England has been found for the period under study [54]. The strengths of this study are the large sample size, including around a quarter of women in the UK in the target age range at recruitment, and the virtually complete follow-up both for hospital admissions and for deaths attributed to CHD. The women in this study included the first generation in the UK in which a considerable proportion have smoked for their entire adult lives and among whom the full effect of smoking could be reliably assessed [29]. Education and area deprivation at recruitment reflect material circumstances both in early life and in middle age, and education in particular was strongly associated with participants’ reported household circumstances in childhood. A further strength of this study is the inclusion of adjustment for the effects of alcohol consumption and physical inactivity on inequalities in CHD risk, which have rarely been examined as possible explanatory factors in previous studies [16].

## Conclusions

In this study of UK women, much of the inequality in CHD risk associated with education and area deprivation was accounted for by smoking, alcohol consumption, physical inactivity and BMI.

## Additional file

Additional file 1: Table S1. Agreement of reported health-related behaviours on identical recruitment questionnaires by education and by area deprivation (*n* = 19,309). **Table S2.** Changes in health behaviours over 3 years by education and by area deprivation. **Table S3.** Relative risks and 95 % confidence intervals (CIs) of coronary heart disease (CHD) incidence by level of education and area deprivation combined. **Table S4.** Relative risks and 95 % CIs of CHD incidence and CHD mortality by education, restricted to never smokers (589,237 women). **Table S5.** Relative risks and 95 % CIs of CHD incidence and CHD mortality by area deprivation, restricted to never smokers (589,237 women). **Table S6.** Relative risks and 95 % CIs of CHD incidence and CHD mortality by area deprivation quintiles, using national deprivation quintiles (1,202,839 women). **Table S7.** Relative risks and 95 % CIs of CHD incidence and CHD mortality by education, excluding women treated for hypertension or diabetes at baseline (1,014,256 women). **Table S8.** Relative risks and 95 % CIs of CHD incidence and CHD mortality by area deprivation quintile, excluding women treated for hypertension or diabetes at baseline (1,014,256 women). **Table S9.** Relative risks and 95 % CIs of CHD incidence and CHD mortality by area deprivation quintile, using a clustered sandwich estimator (1,202,983 women). (DOCX 54 kb)

## Abbreviations

BMI: Body mass index
CHD: Coronary heart disease
LR: Likelihood-ratio χ^2^ statistic
NHS: National health service
