# Supplementary material for: The role of health-related behavioural factors in accounting for inequalities in coronary heart disease risk by education and area deprivation: prospective study of 1.2 million UK women
Source: BMC Med. 2016 Oct 13;14:145. doi: 10.1186/s12916-016-0687-2 (PMC5062936; doi:10.1186/s12916-016-0687-2)
Supplement: Additional file 1: Table S1. — Agreement of reported health-related behaviours on identical recruitment questionnaires by education and by area deprivation (n = 19,309). Table S2. Changes in health behaviours over 3 years by education and by area deprivation. Table S3. Relative risks and 95 % confidence intervals (CIs) of coronary heart disease (CHD) incidence by level of education and area deprivation combined. Table S4. Relative risks and 95 % CIs of CHD incidence and CHD mortality by education, restricted to never smokers (589,237 women). Table S5. Relative risks and 95 % CIs of CHD incidence and CHD mortality by area deprivation, restricted to never smokers (589,237 women). Table S6. Relative risks and 95 % CIs of CHD incidence and CHD mortality by area deprivation quintiles, using national deprivation quintiles (1,202,839 women). Table S7. Relative risks and 95 % CIs of CHD incidence and CHD mortality by education, excluding women treated for hypertension or diabetes at baseline (1,014,256 women). Table S8. Relative risks and 95 % CIs of CHD incidence and CHD mortality by area deprivation quintile, excluding women treated for hypertension or diabetes at baseline (1,014,256 women). Table S9. Relative risks and 95 % CIs of CHD incidence and CHD mortality by area deprivation quintile, using a clustered sandwich estimator (1,202,983 women). (DOCX 54 kb) [file 12916_2016_687_MOESM1_ESM.docx]

**Additional File 1**

**The role of health-related behavioural factors in accounting for inequalities in coronary heart disease risk by education and area deprivation: prospective study of 1.2 million UK women**

Sarah Floud, Angela Balkwill, Kath Moser, Gillian K. Reeves, Jane Green, Valerie Beral, Benjamin J. Cairns, for the Million Women Study Collaborators

**Table S1 – Agreement of reported health-related behaviours on identical recruitment questionnaires by education and by area deprivation (N = 19,309)**

|  |  | **Kappa statistic** | | | | | | |
| --- | --- | --- | --- | --- | --- | --- | --- | --- |
|  |  | **Education** | | |  | **Area deprivation** | | |
|  |  | **Tertiary** | **Secondary/**  **Technical** | **No**  **qualifications** |  | **Least**  **deprived** | **Middle Third** | **Most**  **deprived** |
|  | **No. of women** | 2272 | 7132 | 7371 |  | 5365 | 5651 | 5759 |
| **Health-related behaviour** | **Categories** |  |  |  |  |  |  |  |
| Smoking | never, past, current | 0.90 | 0.90 | 0.89 |  | 0.90 | 0.89 | 0.90 |
| Body mass index (kg/m^2^) | <25, 25-, 30+ | 0.72 | 0.70 | 0.70 |  | 0.71 | 0.71 | 0.69 |
| Alcohol (drinks/wk) | <7, 7-14, 15+ | 0.65 | 0.64 | 0.62 |  | 0.65 | 0.63 | 0.63 |
| Physical activity | rarely/never, <once/wk, >=once/wk | 0.42 | 0.41 | 0.44 |  | 0.44 | 0.43 | 0.44 |

**Table S2 Changes in health behaviours over 3 years by education and by area deprivation**

|  | **Education** | | **Area deprivation** | |
| --- | --- | --- | --- | --- |
|  | **Qualifications** | **No qualifications** | **Least deprived half** | **Most deprived half** |
|  |  |  |  |  |
| **Smoking** |  |  |  |  |
| % of current smokers at baseline who reported being past smokers 3y later | 25 | 23 | 25 | 23 |
| **Alcohol** |  |  |  |  |
| % reporting 7-14 drinks/wk at baseline who reported 15+ drinks/wk 3y later | 13 | 11 | 12 | 12 |
| % reporting 7-14 drinks/wk at baseline who reported <7 drinks/wk 3y later | 22 | 30 | 23 | 27 |
| **Body Mass Index** |  |  |  |  |
| % women <25 kg/m^2^ at baseline who were 25-30 kg/m^2^ 3y later | 14 | 18 | 15 | 16 |
| % women 25-30 kg/m^2^ at baseline who were 30+ kg/m^2^ 3 y later | 9 | 11 | 9 | 11 |
| **Physical activity** |  |  |  |  |
| % women doing any exercise >=once per week at baseline who were in lowest tertile of excess MET-hours† 3y later | 30 | 28 | 28 | 30 |
|  |  |  |  |  |

%’s relate to women who were asked the question on both recruitment and re-survey

† Excess MET-hours: metabolic equivalent hours of energy expenditure in excess of basal metabolic rate (for details see Armstrong ME, Cairns BJ, Green J, Reeves GK, Beral V, Million Women Study C: Reported frequency of physical activity in a large epidemiological study: relationship to specific activities and repeatability over time. BMC Med Res Methodol 2011, 11:97.)

**Table S3 – Relative risks and 95% CIs of CHD incidence by level of education and area deprivation combined**

|  | **Least deprived** | **Middle third** | **Most deprived** | **LR** | **% reduction in LR** |
| --- | --- | --- | --- | --- | --- |
|  | **RR (95% CI)** | **RR (95% CI)** | **RR (95% CI)** |  |  |
| Adjusted for age, birth cohort and region only | |  |  |  |  |
| **Tertiary** | 1.00 (-) | 1.13 (1.06-1.19) | 1.41 (1.32-1.51) |  |  |
| **Secondary/technical** | 1.24 (1.18-1.29) | 1.46 (1.39-1.52) | 1.91 (1.82-2.00) |  |  |
| **No qualifications** | 1.71 (1.64-1.80) | 1.97 (1.88-2.06) | 2.65 (2.54-2.77) | 6202 |  |
|  |  |  |  |  |  |
| Adjusted for age, birth cohort, region, smoking, alcohol, physical inactivity, body mass index | | | | |  |
| **Tertiary** | 1.00 (-) | 1.08 (1.02-1.14) | 1.24 (1.16-1.32) |  |  |
| **Secondary/technical** | 1.14 (1.09-1.19) | 1.27 (1.21-1.33) | 1.49 (1.42-1.56) |  |  |
| **No qualifications** | 1.36 (1.30-1.42) | 1.46 (1.39-1.52) | 1.74 (1.66-1.81) | 1641 | 74 |

LR = Likelihood-ratio test statistic.

Women with tertiary qualifications and in the least deprived quintile are the reference group.

**Table S4 Relative risks and 95% CIs of CHD incidence and CHD mortality by education, restricted to never smokers (589,237 women)**

| **EDUCATION** | **Tertiary** | **Secondary** | **Technical** | **No qualifications** | | **LR†** | **% reduction**  **in LR†** |
| --- | --- | --- | --- | --- | --- | --- | --- |
|  |  |  |  | **Compulsory schooling** | **< Compulsory schooling** |  |  |
| **CHD INCIDENCE**  **No. of first CHD events** | 3057 | 6570 | 4722 | 12 451 | 1017 |  |  |
| **Relative risk (95% CI), adjusted for:** |  |  |  |  |  |  |  |
| -Age, birth cohort and region only | 1.00 (-) | 1.14 (1.09-1.19) | 1.28 (1.23-1.34) | 1.60 (1.54-1.67) | 2.16 (2.01-2.32) | 1026 |  |
| -Age, birth cohort, region, health behaviours‡ | 1.00 (-) | 1.08 (1.04-1.13) | 1.17 (1.12-1.22) | 1.32 (1.26-1.37) | 1.66 (1.55-1.79) | 347 | 66 |
| -Age, birth cohort, region, health behaviours‡, plus deprivation | 1.00 (-) | 1.08 (1.03-1.12) | 1.16 (1.11-1.21) | 1.26 (1.21-1.32) | 1.56 (1.45-1.68) | 241 | 77 |
|  |  |  |  |  |  |  |  |
| **CHD MORTALITY**  **No. of CHD deaths** | 157 | 386 | 256 | 856 | 70 |  |  |
| **Relative risk (95% CI), adjusted for:** |  |  |  |  |  |  |  |
| -Age, birth cohort and region only | 1.00 (-) | 1.31 (1.09-1.57) | 1.29 (1.06-1.58) | 1.96 (1.65-2.32) | 2.56 (1.93-3.39) | 111 |  |
| -Age, birth cohort, region, health behaviours‡ | 1.00 (-) | 1.22 (1.01-1.47) | 1.13 (0.93-1.38) | 1.45 (1.21-1.73) | 1.67 (1.26-2.23) | 29 | 74 |
| -Age, birth cohort, region, health behaviours‡, plus deprivation | 1.00 (-) | 1.21 (1.00-1.46) | 1.12 (0.91-1.37) | 1.35 (1.14-1.62) | 1.50 (1.12-2.00) | 17 | 84 |

† Likelihood-ratio test statistic

‡ Smoking, alcohol consumption, physical inactivity, body mass index

**Table S5 Relative risks and 95% CIs of CHD incidence and CHD mortality by area deprivation, restricted to never smokers (589,237 women)**

| **AREA DEPRIVATION** | **Least deprived quintile** | **Q2** | **Q3** | **Q4** | **Most deprived quintile** | **LR†** | **% reduction**  **in LR†** |
| --- | --- | --- | --- | --- | --- | --- | --- |
| **CHD INCIDENCE**  **No. of first CHD events** | 5264 | 5586 | 5553 | 5774 | 5640 |  |  |
| **Relative risk (95% CI), adjusted for:** |  |  |  |  |  |  |  |
| -Age, birth cohort and region only | 1.00 (-) | 1.11 (1.07-1.15) | 1.18 (1.13-1.22) | 1.36 (1.31-1.41) | 1.71 (1.64-1.77) | 909 | - |
| -Age, birth cohort, region, health behaviours‡ | 1.00 (-) | 1.07 (1.03-1.12) | 1.11 (1.07-1.15) | 1.23 (1.19-1.28) | 1.43 (1.38-1.49) | 399 | 56 |
| -Age, birth cohort, region, health behaviours‡, plus education | 1.00 (-) | 1.06 (1.03-1.11) | 1.09 (1.05-1.13) | 1.20 (1.16-1.25) | 1.37 (1.31-1.42) | 293 | 68 |
|  |  |  |  |  |  |  |  |
| **CHD MORTALITY**  **No. of CHD deaths** | 270 | 359 | 320 | 353 | 423 |  |  |
| **Relative risk (95% CI), adjusted for:** |  |  |  |  |  |  |  |
| -Age, birth cohort and region only | 1.00 (-) | 1.36 (1.17-1.60) | 1.29 (1.10-1.52) | 1.57 (1.34-1.84) | 2.46 (2.11-2.87) | 147 | - |
| -Age, birth cohort, region, health behaviours‡ | 1.00 (-) | 1.30 (1.11-1.52) | 1.18 (1.00-1.38) | 1.34 (1.15-1.58) | 1.87 (1.60-2.19) | 69 | 53 |
| -Age, birth cohort, region, health behaviours‡, plus education | 1.00 (-) | 1.29 (1.10-1.51) | 1.16 (0.98-1.36) | 1.31 (1.11-1.53) | 1.78 (1.52-2.09) | 58 | 61 |

† Likelihood-ratio test statistic

‡ Smoking, alcohol consumption, physical inactivity, body mass index

**Table S6 Relative risks and 95% CIs of CHD incidence and CHD mortality by area deprivation quintiles, using national deprivation quintiles (1,202,839 women)**

| **AREA DEPRIVATION** | **Least deprived quintile** | **Q2** | **Q3** | **Q4** | **Most deprived quintile** | **LR†** | **% reduction**  **in LR†** |
| --- | --- | --- | --- | --- | --- | --- | --- |
| **CHD INCIDENCE**  **No. of first CHD events** | 16 156 | 15 748 | 14 966 | 13 344 | 11 680 |  |  |
| **Relative risk (95% CI), adjusted for:** |  |  |  |  |  |  |  |
| -Age, birth cohort and region only | 1.00 (-) | 1.14 (1.11-1.16) | 1.34 (1.31-1.37) | 1.63 (1.59-1.66) | 2.08 (2.03-2.13) | 4268 | - |
| -Age, region, health behaviours‡ | 1.00 (-) | 1.07 (1.05-1.09) | 1.18 (1.15-1.20) | 1.30 (1.27-1.33) | 1.50 (1.46-1.53) | 1218 | 72 |
| -Age, region, health behaviours‡, plus education | 1.00 (-) | 1.06 (1.03-1.08) | 1.15 (1.12-1.17) | 1.25 (1.22-1.28) | 1.42 (1.38-1.45) | 891 | 79 |
|  |  |  |  |  |  |  |  |
| **CHD MORTALITY**  **No. of CHD deaths** | 1116 | 1225 | 1267 | 1280 | 1144 |  |  |
| **Relative risk (95% CI), adjusted for:** |  |  |  |  |  |  |  |
| -Age, birth cohort and region only | 1.00 (-) | 1.27 (1.17-1.38) | 1.61 (1.49-1.75) | 2.22 (2.05-2.40) | 2.96 (2.72-3.22) | 820 | - |
| -Age, region, health behaviours‡ | 1.00 (-) | 1.14 (1.05-1.23) | 1.28 (1.18-1.39) | 1.48 (1.36-1.61) | 1.63 (1.49-1.78) | 161 | 80 |
| -Age, region, health behaviours‡, plus education | 1.00 (-) | 1.12 (1.04-1.22) | 1.25 (1.16-1.36) | 1.43 (1.32-1.56) | 1.56 (1.42-1.70) | 129 | 84 |

† Likelihood-ratio test statistic

‡ Smoking, alcohol consumption, physical inactivity, body mass index

**Table S7 Relative risks and 95% CIs of CHD incidence and CHD mortality by education, excluding women treated for hypertension or diabetes at baseline (1,014,256 women)**

| **EDUCATION** | **Tertiary** | **Secondary** | **Technical** | **No qualifications** | | **LR†** | **% reduction**  **in LR†** |
| --- | --- | --- | --- | --- | --- | --- | --- |
|  |  |  |  | **Compulsory schooling** | **< Compulsory schooling** |  |  |
| **CHD INCIDENCE**  **No. of first CHD events** | 4360 | 10 393 | 7839 | 26 224 | 1908 |  |  |
| **Relative risk (95% CI), adjusted for:** |  |  |  |  |  |  |  |
| -Age, birth cohort and region only | 1.00 (-) | 1.22 (1.18-1.27) | 1.41 (1.36-1.46) | 1.94 (1.88-2.01) | 2.45 (2.32-2.58) | 3214 |  |
| -Age, birth cohort, region, health behaviours‡ | 1.00 (-) | 1.13 (1.09-1.17) | 1.22 (1.18-1.27) | 1.43 (1.39-1.48) | 1.72 (1.62-1.81) | 825 | 74 |
| -Age, birth cohort, region, health behaviours‡, plus deprivation | 1.00 (-) | 1.12 (1.09-1.17) | 1.22 (1.17-1.26) | 1.37 (1.32-1.42) | 1.61 (1.53-1.70) | 582 | 82 |
|  |  |  |  |  |  |  |  |
| **CHD MORTALITY**  **No. of CHD deaths** | 267 | 695 | 481 | 2052 | 170 |  |  |
| **Relative risk (95% CI), adjusted for:** |  |  |  |  |  |  |  |
| -Age, birth cohort and region only | 1.00 (-) | 1.34 (1.17-1.55) | 1.37 (1.18-1.60) | 2.37 (2.08-2.69) | 3.24 (2.67-3.93) | 400 |  |
| -Age, birth cohort, region, health behaviours‡ | 1.00 (-) | 1.15 (1.00-1.33) | 1.07 (0.92-1.24) | 1.34 (1.17-1.53) | 1.65 (1.36-2.01) | 48 | 88 |
| -Age, birth cohort, region, health behaviours‡, plus deprivation | 1.00 (-) | 1.15 (1.00-1.32) | 1.05 (0.91-1.23) | 1.26 (1.10-1.44) | 1.52 (1.25-1.85) | 28 | 93 |

† Likelihood-ratio test statistic

‡ Smoking, alcohol consumption, physical inactivity, body mass index

**Table S8 Relative risks and 95% CIs of CHD incidence and CHD mortality by area deprivation quintile, excluding women treated for hypertension or diabetes at baseline (1,014,256 women)**

| **AREA DEPRIVATION** | **Least deprived quintile** | **Q2** | **Q3** | **Q4** | **Most deprived quintile** | **LR†** | **% reduction**  **in LR†** |
| --- | --- | --- | --- | --- | --- | --- | --- |
| **CHD INCIDENCE**  **No. of first CHD events** | 8041 | 8743 | 9509 | 10 821 | 13 610 |  |  |
| **Relative risk (95% CI), adjusted for:** |  |  |  |  |  |  |  |
| -Age, birth cohort and region only | 1.00 (-) | 1.11 (1.07-1.14) | 1.24 (1.20-1.28) | 1.46 (1.42-1.50) | 1.97 (1.92-2.03) | 2981 | - |
| -Age, birth cohort, region, health behaviours‡ | 1.00 (-) | 1.05 (1.02-1.08) | 1.13 (1.09-1.16) | 1.23 (1.20-1.27) | 1.45 (1.41-1.49) | 843 | 72 |
| -Age, birth cohort, region, health behaviours‡, plus education | 1.00 (-) | 1.04 (1.01-1.07) | 1.10 (1.07-1.14) | 1.19 (1.16-1.23) | 1.37 (1.33-1.41) | 600 | 80 |
|  |  |  |  |  |  |  |  |
| **CHD MORTALITY**  **No. of CHD deaths** | 461 | 587 | 639 | 798 | 1180 |  |  |
| **Relative risk (95% CI), adjusted for:** |  |  |  |  |  |  |  |
| -Age, birth cohort and region only | 1.00 (-) | 1.29 (1.14-1.46) | 1.44 (1.28-1.62) | 1.86 (1.65-2.08) | 2.99 (2.68-3.33) | 534 | - |
| -Age, birth cohort, region, health behaviours‡ | 1.00 (-) | 1.17 (1.04-1.32) | 1.20 (1.06-1.35) | 1.34 (1.19-1.50) | 1.66 (1.49-1.86) | 101 | 81 |
| -Age, birth cohort, region, health behaviours‡, plus education | 1.00 (-) | 1.16 (1.03-1.31) | 1.18 (1.05-1.33) | 1.30 (1.16-1.46) | 1.60 (1.43-1.79) | 82 | 85 |

† Likelihood-ratio test statistic

‡ Smoking, alcohol consumption, physical inactivity, body mass index

**Table S9 Relative risks and 95% CIs of CHD incidence and CHD mortality by area deprivation quintile, using a clustered sandwich estimator (1,202,983 women)**

| **AREA DEPRIVATION** | **Least deprived quintile** | **Q2** | **Q3** | **Q4** | **Most deprived quintile** |
| --- | --- | --- | --- | --- | --- |
| **CHD INCIDENCE**  **No. of first CHD events** | 11052 | 12309 | 13365 | 15429 | 19742 |
| **Relative risk (95% CI), adjusted for:** |  |  |  |  |  |
| -Age, birth cohort and region only | 1.00 (-) | 1.12 (1.09-1.15) | 1.24 (1.21-1.27) | 1.46 (1.43-1.50) | 1.96 (1.92-2.01) |
| -Age, birth cohort, region, health behaviours‡ | 1.00 (-) | 1.06 (1.04-1.09) | 1.12 (1.09-1.15) | 1.23 (1.20-1.26) | 1.45 (1.42-1.49) |
| -Age, birth cohort, region, health behaviours‡, plus education | 1.00 (-) | 1.05 (1.02-1.08) | 1.10 (1.07-1.13) | 1.20 (1.17-1.23) | 1.38 (1.35-1.41) |
|  |  |  |  |  |  |
| **CHD MORTALITY**  **No. of CHD deaths** | 727 | 965 | 1045 | 1357 | 1938 |
| **Relative risk (95% CI), adjusted for:** |  |  |  |  |  |
| -Age, birth cohort and region only | 1.00 (-) | 1.32 (1.20-1.46) | 1.45 (1.32-1.60) | 1.92 (1.75-2.10) | 2.91 (2.67-3.17) |
| -Age, birth cohort, region, health behaviours‡ | 1.00 (-) | 1.21 (1.10-1.33) | 1.22 (1.11-1.34) | 1.40 (1.28-1.54) | 1.68 (1.54-1.84) |
| -Age, birth cohort, region, health behaviours‡, plus education | 1.00 (-) | 1.20 (1.09-1.32) | 1.20 (1.09-1.32) | 1.37 (1.25-1.50) | 1.61 (1.47-1.76) |

† Likelihood-ratio test statistic

‡ Smoking, alcohol consumption, physical inactivity, body mass index
